# Pentahydroxyscirpene—Producing Strains, Formation In Planta, and Natural Occurrence

**DOI:** 10.3390/toxins8100295

**Published:** 2016-10-14

**Authors:** Elisabeth Varga, Gerlinde Wiesenberger, Philipp Fruhmann, Alexandra Malachová, Thomas Svoboda, Marc Lemmens, Gerhard Adam, Franz Berthiller

**Affiliations:** 1 Center for Analytical Chemistry and Christian Doppler Laboratory for Mycotoxin Metabolism, Department of Agrobiotechnology (IFA-Tulln), University of Natural Resources and Life Sciences, Vienna (BOKU), Konrad-Lorenz-Straße 20, Tulln 3430, Austria; elisabeth.varga@boku.ac.at (E.V.); alexandra.malachova@boku.ac.at (A.M.); franz.berthiller@boku.ac.at (F.B.); 2 Department of Applied Genetics and Cell Biology, University of Natural Resources and Life Sciences, Vienna (BOKU), Konrad-Lorenz-Straße 24, Tulln 3430, Austria; thomas.svoboda@boku.ac.at (T.S.); gerhard.adam@boku.ac.at (G.A.); 3 Institute of Applied Synthetic Chemistry, Vienna University of Technology (VUT), Getreidemarkt 9/163, Vienna 1060, Austria; philipp.fruhmann@tuwien.ac.at; 4 Institute for Biotechnology in Plant Production, Department of Agrobiotechnology (IFA-Tulln), University of Natural Resources and Life Sciences, Vienna (BOKU), Konrad-Lorenz-Straße 20, Tulln 3430, Austria; marc.lemmens@boku.ac.at

**Keywords:** mycotoxins, LC-MS/MS, taxonomy, toxicity, cereals

## Abstract

Trichothecenes are a class of structurally diverse mycotoxins with more than 200 naturally occurring compounds. Previously, a new compound, pentahydroxyscirpene (PHS), was reported as a byproduct of a nivalenol producing *Fusarium* strain, IFA189. PHS contains a hydroxy group at C-8 instead of the keto group of type B trichothecenes. In this work, we demonstrate that IFA189 belongs to the species *Fusarium kyushuense* using molecular tools. Production of PHS in vitro was also observed for several isolates of other *Fusarium* species producing nivalenol. Furthermore, we report the formation of 4-acetyl-PHS by *F. kyushuense* on inoculated rice. Wheat ears of the variety Remus were infected with IFA189 and the in planta production of PHS was confirmed. Natural occurrence of PHS was verified in barley samples from the Czech Republic using a liquid chromatographic-tandem mass spectrometric method validated for this purpose. Toxicity of PHS to wheat ribosomes was evaluated with a coupled in vitro transcription and translation assay, which showed that PHS inhibits protein biosynthesis slightly less than nivalenol and deoxynivalenol.

## 1. Introduction

**Figure 1:**
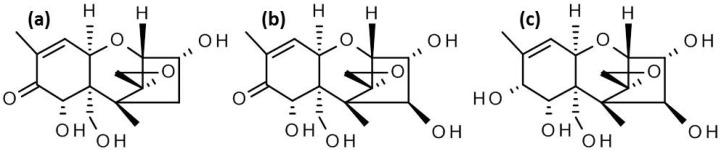
Chemical structures of (**a**) deoxynivalenol; (**b**) nivalenol; and (**c**) pentahydroxyscirpene.

Mycotoxins are secondary metabolites of molds, which are toxic to humans and animals. While the Food and Agriculture Organization of the United Nations (FAO) estimated in the 1990s that about a quarter of all agricultural commodities worldwide are significantly contaminated with mycotoxins [1], more recent findings suggest an even higher rate [2]. In temperate regions of the world, *Fusarium* spp. are the most commonly occurring toxigenic fungi on crops. Thereof, the most important species is *Fusarium graminearum*, which can infect a wide range of cereals including wheat, barley, oats, rye, or maize [3]. Species within the *F. graminearum* complex can produce type B trichothecenes in planta [4,5]. Based on the different ability to hydroxylate C-4 either deoxynivalenol (DON, Figure 1a.) or nivalenol (NIV, Figure 1b) chemotypes are distinguished. DON producing strains of *F. graminearum* contain a loss of function allele of the *TRI13* (cytochrome P450) gene [6]. Members of the DON chemotype are usually subdivided into 3-acetyl-deoxynivalenol (3ADON) and 15-acetyl-deoxynivalenol (15ADON) strains, based on which acetylated precursor accumulates in vitro, caused by allelic differences in the *TRI8* encoded esterase [7,8]. In most NIV strains 4-acetylnivalenol (fusarenon X, FUSX) co-occurs. During plant infection the acetyl groups are also removed by plant carboxylesterases [9]. Trichothecenes are chemically and thermally very stable and are readily carried over from raw cereals into processed food (e.g., [10]).

In sensitive species, e.g., humans or pigs, type B trichothecenes elicit anorexia, abdominal distress, malaise, diarrhea, emesis, impaired weight gain, gastroenteritis, and immunotoxicity (reviewed by [11]). While the major mode of action is the binding of the toxins to eukaryotic ribosomes and interference with protein translation, also intracellular protein kinases are activated, which mediate both selective gene expression and apoptosis (reviewed by [12]). In 2010, the Joint FAO/World Health Organization (WHO) Expert Committee on Food Additives (JECFA) decided to convert the provisional maximum tolerable daily intake (PMTDI) for DON to a group PMTDI of 1 μg/kg body weight (b.w.) for DON and its acetylated derivatives (3ADON and 15ADON) [13]. Likewise, the European Food Safety Authority (EFSA) Panel on Contaminants in the Food Chain (CONTAM) established a tolerable daily intake (TDI) value of 1.2 μg/kg b.w. for NIV [14]. To cope with the threat for consumer safety, maximum levels in food are enforced for DON in many countries—including the European Union [15]—while currently no regulations for NIV are in force.

Only recently a novel mycotoxin, pentahydroxyscirpene (PHS, Figure 1c), which is structurally closely related to NIV was identified [16]. PHS was formed upon inoculation of rice with a NIV producing *Fusarium* strain in a level of roughly 10% of that of NIV. It was proposed that NIV and PHS share the common precursor 7,8-dihydroxy-3,4,15-triacetoxyscirpenol, which leads to the formation of PHS if the function of (a yet unknown) oxidoreductase is impaired. This intermediate is excreted and deacetylated before the keto group of NIV is formed. The occurrence and relevance in plant pathogen interaction and toxicological significance of this compound is yet unknown.

In this study, the molecular identification of the PHS producer IFA189 is reported and other selected NIV-producing *Fusarium* strains and species were characterized regarding the ability to form PHS. We furthermore investigated the in vitro toxicity of PHS to wheat ribosomes and whether PHS is formed in planta.

## 2. Results

### 2.1. Development and Validation of the Liquid Chromatographic-Tandem Mass Spectrometric (LC-MS/MS) Method

Mass spectrometric parameters were optimized for the target compounds (PHS, NIV, FUSX) using syringe injection of single analyte solutions and are summarized in Table 1. All analytes showed higher signal intensities in negative electrospray ionization mode. In case of PHS the deprotonated ion was chosen as precursor, whereas for the other two compounds the acetate adducts showed the highest signal intensity. While PHS is eluting quite early under reversed phase chromatographic conditions, a retention factor k’ of approximately 1.2 could be achieved with selecting 5% methanol (MeOH) as the starting mobile phase for the linear gradient.

The method was validated for barley by spiking blank samples with PHS and NIV before extraction (with two different solvents) on five different levels in triplicate and after extraction on one level in five replicates. For the acetonitrile (ACN) based extraction, the determined apparent recoveries were 137% for PHS and 98% for NIV. A significant signal enhancement (174% for PHS and 114% for NIV) was observed. While rather uncommon for (more apolar) type B trichothecenes, the signal enhancement for the very polar PHS is seemingly caused by early eluting matrix components. The resulting extraction recoveries were 79% and 86% for PHS and NIV respectively, and relative standard deviations of less than 11% were achieved. Very similar results were obtained using the more polar MeOH based extraction method. There, the extraction recoveries were 103% and 91% for PHS and NIV, while the apparent recoveries were 173% for PHS (due to matrix effects) and 99% for NIV. Relative standard deviations were about 10% for both analytes. The suitability of the used acidified ACN:water mixture to also extract a variety of other mycotoxins [17] resulted in the preferred usage of this solvent.

### 2.2. Molecular Identification of IFA189 as *Fusarium kyushuense*

The PHS producing strain IFA189 had initially been received as *Fusarium sporotrichioides* suitable for NIV production (see discussion). To clarify its taxonomic status, we used the “Fusarium ID” approach described by Geiser et al. [18]. Part of Translation Elongation Factor 1α (TEF1α) was amplified using primers ef1 and ef2. The resulting polymerase chain reaction (PCR) product was sequenced with nested primers EF15fw and EF16rev [19] and the resulting sequence was blasted against sequences deposited at the Fusarium ID database [20] and at the National Center for Biotechnology Information [21]. A fragment of 646 base pairs (bp) showed 99.84% identity to TEF1α from *F. kyushuense* at Fusarium ID, while the blast search at NCBI revealed 100% identity to a 668 bp fragment form TEF1α of the same species (*Fusarium kyushuense* NRRL 6490, accession number AB674297.1). Thus we conclude that strain IFA189 is *Fusarium kyushuense*.

### 2.3. PHS Production by Other NIV Producers

NIV was first isolated from the “*Fusarium nivale*” strain Fn-2B (sometimes also termed Fn2B or FN-2B in various references), which eventually was recognized to be *F. kyushuense* (see conclusions below). The rice material used for feeding trials was obtained using this strain and also NIV for toxicological studies was purified from it. We therefore obtained all available *F. kyushuense* strains from the Agricultural Research Service (ARS) Culture Collection (NRRL) strain collection and tested them for PHS production. We furthermore set out to test whether NIV producing isolates from other species also produce significant amounts of PHS. Different known and suspected (based on genotyping) NIV-producing strains belonging to the genus *Fusarium* (*F. asiaticum*, *F. culmorum*, *F. equiseti*, *F. graminearum,* and *F. kyushuense*) from our strain collections were selected for the screening of PHS. The taxonomic classification of all used strains was confirmed by TEF1α sequencing. In extracts from five *F. graminearum* and one *F. equiseti* isolates the presence of PHS was confirmed, but at a far lower scale than in *F. kyushuense* extracts (Table 2). Also, three of the six *F. kyushuense* strains from the NRRL collection produced PHS on autoclaved rice.

In one paper, it is claimed that *F. kyushuense* contains aflatoxin biosynthesesis genes and is able to produce aflatoxins [23]. Using the published primer sequences we were unable to obtain the described nor-PCR product in IFA189 or the strain used in the publication (NRRL 3509). At low stringency PCR fragments of the expected size (300 bp) were produced, isolated, and cloned into the vector pCR^®^4-TOPO^®^. DNA sequences from 12 transformants contained inserts with no homology to the nor1 gene from any *Aspergillus* species. Using LC-MS/MS analysis, neither aflatoxin B_1_ nor aflatoxin G_1_ were detected in extracts of the cultures (detection limit of the method 0.05 µg/kg), thus disproving the previous report.

### 2.4. Tentative Identification of 4-Acetyl-Pentahydroxyscirpene

**Figure 2:**
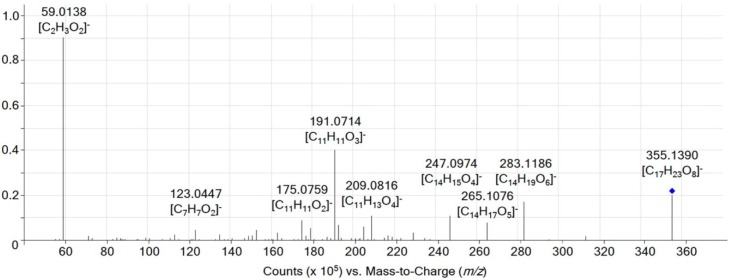
High resolution tandem mass spectrometric product ion scan of the tentatively identified 4-acetyl-pentahydroxyscirpene in negative electrospray ionization mode at a collision energy of 15 eV.

As evident from Table 2, most strains produced more FUSX than NIV under the conditions used. FUSX, the presumed biosynthetic precursor of NIV, differs from NIV only by an acetyl-group at C-4. Likewise an acetylated form of PHS is likely to exist (see also Scheme 2 in [16]). Extracts of the *F. kyushuense* IFA189 rice cultures were measured with ultra-high performance liquid chromatography (UHPLC) coupled to a quadrupole time-of-flight mass spectrometer (QTOF). The mass spectrum of a peak at 6.35 min showed ions with *m*/*z* 355.1396 and *m*/*z* 401.1448. These ions closely match with the calculated [M − H]^−^ ion (*m*/*z* 355.1398, ∆*m* = −0.6 ppm) and the [M + HCOO]^−^ (*m*/*z* 401.1453, ∆*m* = −1.2 ppm) ion of a C_17_H_24_O_8_ compound, thus verifying the expected sum formula. Tandem mass spectrometric measurements supported the hypothesis of a PHS metabolite since fragments of acetyl-PHS (Figure 2) matched those of PHS (see supporting information of [16]). The site of conjugation cannot be pinpointed by LC-MS/MS alone, but equally as with FUSX, the C-4 position seems very likely. Due to the lack of a standard, no absolute quantification of the identified acetyl-PHS was possible.

### 2.5. PHS-Impurity in Commercial NIV-Standards

Seemingly *Fusarium* strains able to produce (high concentrations of) NIV, also produce PHS. As the polarity of both substances is similar, it is conceivable that purified nivalenol standards might also contain PHS. Purity determination might be compromised by the low absorbance of PHS (no conjugated double bond) as well as the similar molecular mass (difference of 2 amu) and structure. Commonly used methods for purity determination of natural toxins, like type B trichothecenes, include LC-UV methods, nuclear magnetic resonance (NMR), or elemental analysis [24]. Given the nature of the compound, it is likely that those methods will fail to identify PHS as impurity in NIV. Therefore, we acquired commercially available NIV standards (solid or calibrants) to determine PHS. The certified reference material IRMM-316 (liquid calibrant) contained 0.2% PHS in relation to NIV. In the liquid calibrants obtained from Romer Labs from 2006 to 2012, up to 20.8% PHS were detected, whereas all older and newer NIV-calibrants contained only up to 0.2% PHS. It has to be mentioned though that, despite the contamination with PHS, the measured concentration of NIV in all liquid calibrants from Romer Labs was within 98%–102% of the indicated NIV concentration. The IRMM standard, however, seemingly contained only 92% of the certified concentration. In the solid nivalenol hydrate standard (with an indicated purity of 98%) purchased from Santa Cruz (Santa Cruz, CA, USA) actually 3.7% PHS were quantified, while in solid NIV standards from *F. nivale* obtained from Sigma Aldrich (Vienna, Austria)(purity ≥ 98%) less than 0.1% PHS were determined by LC-MS/MS measurements.

### 2.6. Natural Occurrence of PHS

As a first step to test whether PHS is also produced in planta and might occur naturally, we inoculated wheat ears of the Fusarium susceptible variety “Remus” [25] at anthesis by injecting a spore suspension of strain IFA189 into two outer florets. After five weeks, the ears were visually inspected. The observed symptoms were much weaker than with *F. graminearum* and only local infection of single spikelets expressed as brown necrotic lesions were visible. Similar necrotic lesions were visible after the treatment of the wheat ears with solutions of NIV or PHS. A wheat ear treated with the spore suspension culture was milled, extracted, and measured with the LC-MS/MS method. The determined concentrations were 1.8 mg/kg for PHS and 12 mg/kg for NIV resulting in a PHS to NIV-ratio of 0.15.

Subsequently, we re-analyzed naturally infected grain samples with high NIV concentrations. The presence of PHS in naturally contaminated samples was for the first time confirmed in barley samples from Kromeriz (a town in the Zlin region of the Czech Republic). The three positive samples contained 51 µg/kg PHS and 0.8 mg/kg NIV (PHS/NIV ratio 0.06), 65 µg/kg PHS and 1 mg/kg NIV (PHS/NIV ratio 0.06), as well as 310 µg/kg PHS and 4.6 mg/kg NIV (PHS/NIV ratio 0.07).

### 2.7. Toxicity of PHS to Wheat Ribosomes

**Figure 3:**
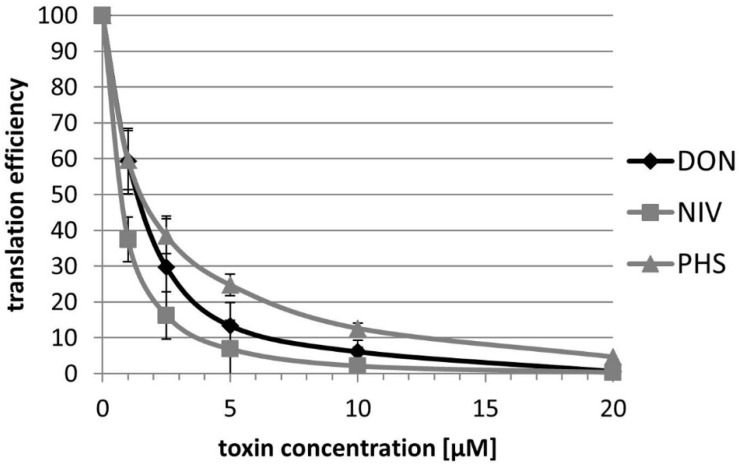
Inhibition of in vitro translation by DON, NIV, and PHS.

Trichothecenes bind to the large subunit of eukaryotic ribosomes and inhibit translation [26]. To test the toxicity of PHS towards plant ribosomes we performed in vitro translation experiments using a commercial wheat germ extract (Figure 3). The IC_50_ values for NIV, DON, and PHS are 0.75, 1.4, and 1.5, respectively. Therefore, we conclude that PHS efficiently inhibits translation and is only slightly less toxic than DON or NIV for plant ribosomes and thus is likely to contribute to virulence.

## 3. Discussion

Strain IFA189 was received about 20 years ago, at that time classified as *F. sporotrichioides* suitable for the production of FUSX and NIV (Prof. H. Pettersson, personal communication). Molecular identification revealed that it actually is a *F. kyushuense* strain, and most likely identical to Fn-2B [27]. The toxic principle nivalenol was isolated for the first time in 1968 [28] from isolate Fn-2B, at that time designated “*Fusarium nivale*” (*F. nivale* is now *Microdochium nivale* and considered to be non-toxinogenic [29]). Strain Fn-2B has a changeful taxonomic history. In 1984, it was described as *Fusarium tricinctum* producing NIV and FUSX [30]. It is deposited as NRRL 6490 (and backup NRRL 25348). IFA189 and these two strains produced the highest levels and proportion of PHS in our hands (see Table 2). In the monography of “Toxigenic *Fusarium* species” Fn-2B [31] was classified as *F. sporotrichioides*. Then, based on partial (low quality) ribosomal RNA sequences, it was proposed to be close to *Fusarium poae* in 1995 [32]. Eventually, in 1998 the new species *Fusarium kyushuense* was established, to which this strain belongs according to current molecular taxonomy [33].

The early toxicological experiments with NIV were performed with material derived from strain Fn-2B [34,35,36]. Potentially, PHS was present in a few percent, but this would have only slightly confounded the toxicological results, as PHS has, at the level of the ribosomal target, very similar effects as NIV both in animals and plants based on in vitro translation systems ([16] and this study). However, it is unknown if relevant differences between NIV and PHS in adsorption, metabolism, and excretion exist in animals. For some time, it was controversial whether nivalenol (or material co-eluting with NIV) is genotoxic, causing induction of chromosome aberrations and DNA damage (for review see: [14]). We now can exclude that contamination with aflatoxin—supposedly produced by *F. kyushuense* strains—is a cause for this, as previously claimed [23]. We were unable to detect aflatoxins, both in IFA189 and in NRRL 3509, the strain for which aflatoxin production had been reported. Furthermore, neither strain yielded a nor-1 specific PCR fragment. In agreement with these results, no aflatoxin biosynthesis genes were found in the unpublished whole genome sequence of the *F. kyushuense* strain NRRL 25348 (Robert Proctor, personal communication).

According to the information from the strain collection, NRRL 6491 (=old Fn-M) is producing FUSX, which we could confirm. Yet, *F. kyushuense* strains, e.g., NRRL 3510, are known, which produce primarily the type A trichothecenes T-2 toxin, HT-2 toxin and neosolaniol. In our hands, only a very low level of NIV was detected in extracts of this and other strains (Table 2). Potentially, in the *F. kyushuense* population, strains might co-exist that either hydroxylate only C-8 (either possessing a *TRI1* product like *F. sporotrichioides* [37], or a TRI1p that hydroxylates initially both C-7 and C-8 [38]). The C-8 hydroxy group is subsequently converted to the keto group in NIV or remains a hydroxy group in case of PHS. To produce PHS, this last step must be slow so that the acetylated intermediate can be excreted and deacetylated. Potentially, a variable mix of only C-8 or C-7 & C-8 hydroxylated trichothecenes might be produced. NRRL 3510 produces only minor amounts of NIV, while the NIV-producers make hardly any T-2 toxin, which does not support this speculation. Yet, strains are known which are able to simultaneously produce type A and B trichothecenes, as described for individual isolates of *F. poae* that can co-produce NIV and T-2 toxin plus neosolaniol [39,40] and potentially also PHS. Such “flexible” strains may have an advantage on highly variable host populations, as type B trichothecenes can form Michael adducts with glutathione, while type A trichothecenes lacking the conjugated keto-double bond cannot [41,42]. We could show that PHS is not only produced in culture, but also in planta. As NIV and PHS have similar toxicity for wheat ribosomes, there is seemingly only a small fitness price to pay for the potential advantage that the virulence factor produced by the pathogen can escape glutathione mediated detoxification, if PHS is formed rather than NIV. In this study, we have also presented evidence for the existence of the proposed 4-acetyl-PHS. This is expected if—like in case of NIV—the removal of the C-3 and C-15 acetyl group is faster than removal of the C-4 acetate, as in case of FUSX.

The finding that besides *F. kyushuense* also other high level NIV producing strains may generate a few percent of PHS as byproduct indicates that the toxicological burden of NIV contaminated grain may be slightly higher than anticipated. Our study also shows that PHS can occur in naturally infected grain. Yet this effect is small and toxicologically most likely not relevant. NIV standards may be contaminated with a few percent PHS, which could potentially lead to a small systematic error. Yet, due to increasing awareness, hopefully PHS contamination of calibrants will be soon a matter of the past.

## 4. Materials and Methods

### 4.1. Chemicals and Reagents

MeOH and ACN (both LC gradient grade), as well as glacial acetic acid (p.a.) were purchased from VWR International GmbH (Vienna, Austria) and water was purified using a Purelab Ultra system (ELGA, LabWater, Celle, Germany). Ammonium formate was obtained as a 5 M aqueous solution from Agilent Technologies (Waldbronn, Germany). Ammonium acetate, formic acid (both LC-MS grade), crystalline NIV-standards from *Fusarium nivale* (#N-7769; discontinued) and certified reference material IRMM-316 (NIV in ACN; certified value 24.0 ± 1.1 µg/g; indicative value: 18.8 ± 0.9 µg/mL) were obtained from Sigma Aldrich (Vienna, Austria). Standards for NIV (10.1 µg/mL) and FUSX (10.2 µg/mL) were purchased as a mixed solution of type A and B trichothecenes as well as zearalenone (#002002) in ACN from Romer Labs GmbH (Tulln, Austria). The same supplier also delivered a certified NIV calibrant (100 µg/mL) in ACN (#002011). Solid nivalenol hydrate (sc-236183) was obtained from Santa Cruz Biotechnology Inc. (Santa Cruz, CA, USA), weighted in using a Sartorius M500 P microbalance (Göttingen, Germany) and dissolved in MeOH to obtain a 1000 mg/L solution. PHS was previously purified and characterized by one- and two-dimensional nuclear magnetic resonance spectroscopy, as well as X-ray single-crystal diffraction [16].

### 4.2. LC-MS/MS Parameters

Analyses were performed on a 1290 UHPLC system (Agilent Technologies, Waldbronn, Germany) coupled to a QTrap 5500 mass spectrometer (Sciex, Foster City, CA, USA). For chromatographic separation a Gemini C18 (150 × 4.6 mm, 5 µm) reversed phase column equipped with a guard column (4 × 3 mm) (both from Phenomenex, Aschaffenburg, Germany) were used at 25 °C. The eluents were composed of MeOH:water (eluent A: 5:95, *v*:*v*; eluent B: 98:2, *v*:*v*) and both contained 5 mM ammonium acetate. The flow rate was 1 mL/min and the default injection volume was 3 µL. After an initial holding time at 0% B for 1 min, a linear increase to 85% B within the next 6 min followed. Thereafter, the column was flushed with 100% B for 3 min, followed by re-equilibration for 2.4 min with the starting conditions.

The QTrap 5500 was equipped with a TurboV ion spray source and was operated in negative electrospray ionization mode. The following parameters were used: curtain gas 30 psi (207 kPa, nitrogen), collision gas (nitrogen) medium settings, ion spray voltage −4500 V, source temperature 550 °C, ion source gas 1 and 2 both 80 psi (552 kPa, zero grade air). Optimization of the MS/MS parameters was performed by syringe injection of single analyte solutions. Two (NIV, FUSX) or three (PHS) mass transitions were chosen in the selected reaction monitoring mode and the dwell time was set to 25 ms. The LC-MS instrument was controlled by Analyst software version 1.5.2 (Sciex, Foster City, CA, USA) and automatic and manual integration of the peaks were performed using the same (or higher) software version. Further data processing was carried out in Microsoft Excel 2010.

For high resolution mass spectrometric measurements, a 1290 UHPLC system coupled to a 6550 iFunnel QTOF (both Agilent Technologies (Waldbronn, Germany)) were used. Eluents were water and MeOH, both containing 0.1% formic acid and 1 mM ammonium formate. Chromatographic separation was performed on a Zorbax SB-C18 (150 × 2.1 mm, 1.8 µm, Agilent Technologies) equipped with a C18 UHPLC-guard column from Phenomenex at 30 °C. Starting conditions (10% B) were held for 0.5 min, afterwards a linear gradient to 35% B till 6 min was applied, followed by a wash step with 100% for 1 min and re-equilibration with the starting conditions for 2 min till the end of the run at 9 min. The gas and the sheath gas temperature were 130 °C and 300 °C, respectively, the drying gas flow was 16 L/min and sheath gas flow 11 L/min. The nebulizer was set to 30 psig and the capillary voltage and nozzle voltages were 4 kV and 0.5 kV. Reference masses (*m*/*z* 112.9855 and 966.0007) were used to ensure the high mass accuracy. The mass range was *m*/*z* 100–1000 for MS and *m*/*z* 50–400 for MS/MS measurements and the acquisition rate was three spectra/s for both measurement modes. Instrument control and data evaluation was performed with MassHunter Workstation Software Version B.06.01 and MassHunter Qualitative Analysis Version B.07.00 (both Agilent Technologies, Waldbronn, Germany).

### 4.3. Sample Preparation and Method Validation

Two different extraction solvents were evaluated for the extraction of cereals. ACN:water:acetic acid (79:20:1, *v*:*v*:*v*), an extraction solvent which is often used in multi-mycotoxin analysis [43] was tested as well as a more polar mixture of MeOH:water (50:50, *v*:*v*). For the final method, the following procedure was applied: Cereal samples were ground using an Osterizer Blender (Sunbeam Oster Household Products, Boca Raton, FL, USA) and 5.00 ± 0.01 g of the homogenized sample were weighed into 50-mL polypropylene tubes (Sarstedt, Nümbrecht, Germany). After adding 20 mL of extraction solvent, the samples were briefly shaken by hand followed by an extraction for 60 min at room temperature on a rotary shaker (GFL3017, Burgwedel, Germany). Thereafter, the samples were allowed to settle for a few minutes and an aliquot of the clear extract (500 µL) was diluted with the same amount of water in an HPLC vial.

For method validation, PHS and NIV were spiked before extraction on five different levels (ranging from 10 to 1000 µg/kg) in triplicate and at 12.5 µg/L (equivalent to 100 µg/kg) after extraction in five replicates. A blank barley sample previously checked for the absence of the target analytes was used for the spiking experiments. Spiking before extraction was performed with a mixed solution in MeOH and the samples were stored overnight at room temperature to ensure the evaporation of the solvent. Thereafter, the samples were worked up together with a blank barley sample according to the sample preparation procedure described above. For the preparation of matrix spikes, the extract of the blank barley samples was spiked with the target analytes and diluted with water to achieve the same solvent composition as the samples. Furthermore, separate neat solvent standards (MeOH:water, 50:50, *v*:*v*) were prepared covering a concentration range from 0.38 to 250 µg/L (equivalent to 3 to 2000 µg/kg).

### 4.4. Molecular Classification of the Strain IFA189

For preparation of genomic DNA, we applied the CTAB extraction method [44] with minor modifications. Amplification of EF1α was performed as described in [18]. For sequencing reactions primers EF15fw and EF16rev [19] were used.

To investigate, whether strain IFA189 contains the aflatoxin-biosynthesis gene nor1, we utilized the primers nor_fw and nor_rev [23] for PCR-mediated amplification. The published conditions did not yield a PCR fragment of the expected size (300 bp). Therefore, we applied gradient PCR, which resulted in a 300 bp fragment. We isolated the fragment, cloned it into pTOPO, and prepared the plasmid DNA from 12 transformants, which were subsequently sequenced using the nor primers used for amplification.

### 4.5. Screening for PHS in Naturally Contaminated Cereals and in Known NIV Producing Strains

As PHS is likely to co-occur with the structurally related NIV, cereal samples measured positive for NIV with a previously published method [17] have been re-measured for PHS occurrence. Briefly, maize samples were originating from Malawi, Cameroon, Germany and Austria. Barley samples from an experimental field were provided by Prof. Radim Cerkal from the Czech Republic National Agency for Agricultural Research.

Furthermore, known or suspected NIV producing strains were cultivated as follows: The strains were grown on Fusarium minimal medium (FMM; 1 g/L KH_2_PO_4_, 0.5 g/L MgSO_4_ • 7 H_2_O, 0.5 g/L KCl, 2 g/L NaNO_3_, 30 g/L sucrose, 10 mg/L citric acid, 10 mg/L ZnSO_4_ • 6 H_2_O, 2 mg/L Fe(NH_4_)_2_(SO_4_)_2_ • 6 H_2_O, 0.5 mg/L CuSO_4_ • 5 H_2_O, 0.1 mg/L MnSO_4_, 0.1 mg/L H_3_BO_4_, 0.1 mg/L Na_2_MoO_4_ • 2 H_2_O) from [45] or potato dextrose agar (PDA, Sigma-Aldrich, Vienna, Austria) plates. For the preparation of solid rice medium, 200-mL jars were filled with ca. 10 g rice from a local store and 10 mL reverse osmosis water. The jars were incubated at room temperature for 1 h, before being autoclaved for 60 min at 121 °C. Small agar blocks (approx. 5 × 5 mm) of the FMM or PDA plates with mycelium were transferred to the solid rice medium. The cultures were incubated for two weeks at 20 °C in the dark and then stored at −20 °C until further processing. For extraction, 40 mL of MeOH-water (50:50, *v*:*v*) was added to each of the rice glasses, homogenized using an Ultra-Turrax T25 (IKA-Werke, Staufen, Germany), and extracted for 60 min on a GFL rotary shaker (Burgwedel, Germany). Homogenates were transferred to 50-mL polypropylene tubes and centrifuged (10 min at room temperature, 3200 g). An aliquot of each sample was transferred to 1.5-mL tubes and centrifuged again for 15 min at 20000× *g*, thereafter 800 µL of the supernatants were transferred to HPLC vials for analysis. If required, further dilutions were performed with MeOH-water (50:50, *v*:*v*).

To determine the presence or absence of aflatoxins, *F. kyushuense* strains IFA189 and NRRL 3509 were cultivated on rice. They were first sporulated in mung bean medium, which was prepared as follows: 10 g mung beans were added to 450 mL boiling water and cooked for 20 min. After removal of the mung beans, the extract was filtrated through a folded filter, filled up to 1 L and autoclaved for 20 min at 121 °C. Conidia were separated from mycelia by filtration through a glass-wool filter and sedimented overnight at 4 °C. After removal of the medium spores were re-suspended in water and counted in a Fuchs-Rosenthal chamber. Rice cultures in jars (see above) were inoculated with 10^5^ spores and cultivated both under light (20 °C/55% humidity/24 h light) and dark (20 °C/24 h dark) conditions in triplicate for two weeks. Sample preparation was conducted as described above, except that ACN:water:acetic acid (79:20:1, *v*:*v*:*v*) was used for extraction. To achieve reasonably low limits of detection, extracts were purified with a MycoSep #226 column (Romer Labs). Afterwards, 4 mL of the clean extracts were evaporated to dryness under a gentle stream of nitrogen, re-dissolved in 1 mL MeOH-water (50:50, *v*:*v*), and transferred into HPLC vials. These samples were measured with a multi-mycotoxin-method [17].

### 4.6. Plant Experiments

Spring wheat ears of the German variety “Remus” (“Sappo”/”Mex”/”Famos”) were grown in a glass house using standard conditions [46]. At anthesis, the ears were treated with a spore suspension of *F. kyushuense* containing 5 × 10^5^ conidia/mL. The suspension was injected into the spikelets (10 µL/spikelet) using a syringe and 10 to 15 spikelets were treated per wheat ear. After treatment, the ears were covered with plastic bags for 24 h to provide a high relative air humidity and promote infection after inoculation. Five weeks after the treatment the wheat ears were harvested, frozen, and stored at −20 °C until further processing. Furthermore, wheat ears of the same variety were treated at anthesis with solutions of either NIV or PHS, again harvested after ripening and frozen at −20 °C. Each frozen wheat ear was ground into a fine powder in liquid nitrogen using a mortar. After homogenization of the samples, they were extracted with the fourfold amount of MeOH:water (50:50, *v*:*v*) for 90 min at room temperature, centrifuged, transferred to an HPLC-vial and measured with LC-MS/MS.

### 4.7. Toxicity Assays

In vitro translation assays with TnT^®^ T7 Coupled Wheat Germ Extract System (from Promega (Madison, WI, USA)) were used as described in [47]. Seven independent assays were performed with 12 or 8 individual dilutions of PHS and NIV, respectively.

## Figures and Tables

**Table 1:** List of analytes with optimized mass spectrometric parameters.

Analyte	Retention Time (min)	*m*/*z* Precursor Ion (Declustering Potential in V)	Product Ions (Collision Energy in eV)
Pentahydroxyscirpene	4.0	313.1 (−110)	163.1 (−39)
			175.0 (−30)
			191.1 (−21)
Nivalenol	4.8	371.1 (−75)	59.1 (−42)
			281.1 (−22)
Fusarenon X	6.1	413.2 (−70)	59.1 (−44)
			263.0 (−22)

**Table 2:** Screening for pentahydroxyscirpene (PHS) production of different *Fusarium* strains. Confirmed PHS producing strains are shown in bold letters.

*Fusarium* Strains	PHS (mg/kg)	NIV (mg/kg)	FUSX (mg/kg)
*F. asiaticum* SCK04 (#1) ^1,2^	<0.15	4.84	67.0
*F. asiaticum* SCK04 (#1, PDA) ^3^	<0.15	5.24	42.4
*F. asiaticum* SCK04 (#2) ^2^	<0.15	2.65	12.1
*F. asiaticum* SCK04 (#2, PDA) ^3^	<0.15	3.26	20.8
*F. culmorum* IFA450	<0.15	0.15	0.74
*F. equiseti* IFA33	<0.15	0.23	0.86
*F. equiseti* IFA34	<0.15	<0.012	<0.04
*F. equiseti* IFA63	<0.15	0.07	0.16
*F. equiseti* IFA64	<0.15	0.28	1.23
*F. equiseti* IFA157	<0.15	<0.012	0.05
*F. equiseti* IFA336	<0.15	<0.012	<0.04
*F. equiseti* IFA408	<0.15	0.036	0.15
*F. equiseti* IFA409	<0.15	0.10	0.33
***F. equiseti* IFA410**	**0.23**	**60.0**	**110**
*F. graminearum* DAGZ5	<0.15	0.67	20.0
***F. graminearum* DAGZ8, gray mycelium** ^4^	**0.94**	**294**	**580**
*F. graminearum* DAGZ8, pink mycelium ^4^	<0.15	5.00	212
***F. graminearum* DAGZ13**	**0.17**	**212**	**>6000**
***F. graminearum* DAGZ13 (PDA) ^3^**	**0.29**	**238**	**>5000**
*F. graminearum* DAGZ22	<0.15	0.37	43.6
*F. graminearum* DAGZ23	<0.15	2.71	49.6
***F. graminearum* DAGZ24**	**0.49**	**88.8**	**788**
*F. graminearum* DAGZ25	<0.15	0.10	4.84
*F. graminearum* DAGZ29	<0.15	10.3	242
*F. graminearum* DAGZ31	<0.15	44.0	107
*F. graminearum* DAGZ36	<0.15	0.25	21.9
*F. graminearum* DAGZ37	<0.15	1.91	33.4
*F. graminearum* DAGZ39	<0.15	0.14	1.68
***F. graminearum* DAGZ46**	**1.44**	**399**	**1370**
*F. graminearum* DAGZ47	<0.15	7.72	820
***F. graminearum* DAGZ50 (#1) ^2^**	**1.65**	**199**	**283**
***F. graminearum* DAGZ50 (#2) ^2^**	**1.04**	**359**	**>1000**
***F. graminearum* DAGZ50 (PDA) ^3^**	**2.93**	**>900**	**>3000**
*F. graminearum* DAGZ55	<0.15	32.2	378
*F. graminearum* DAGZ62	<0.15	13.4	133
*F. graminearum* DAGZ63 (#1) ^2^	<0.15	<0.012	16.0
*F. graminearum* DAGZ63 (#1, PDA) ^2,3^	<0.15	0.32	0.568
*F. graminearum* DAGZ63 (#2) ^2^	<0.15	0.06	0.94
*F. graminearum* NRRL 26752	<0.15	1.46	2.42
*F. graminearum* NRRL 26752 (PDA) ^3^	<0.15	1.26	1.48
***F. kyushuense* IFA189 (#1) ^2^**	**110**	**1370**	**1980**
***F. kyushuense* IFA189 (#2) ^2^**	**22.7**	**536**	**4560**
*F. kyushuense* NRRL 3509	<0.15	<0.012	<0.04
*F. kyushuense* NRRL 3510	<0.15	0.03	0.11
***F. kyushuense* NRRL 6490 (=Fn-2B)**	**10.0**	**404**	**2890**
***F. kyushuense* NRRL 6491**	**3.57**	**468**	**2930**
***F. kyushuense* NRRL 25348 (backup of 6490)**	**15.0**	**328**	**1440**
*F. kyushuense* NRRL 26204	<0.15	0.07	0.15

NIV—nivalenol; FUSX—fusarenon-X; ^1^ according to Kim et al. [22]; ^2^ two isolates (#1, #2) of the same strain were available; ^3^ PDA: potato dextrose agar, all other strains were originally cultivated on Fusarium minimal medium (FMM); ^4^ fungus on FMM medium was either grayish with air mycelium or reddish-pink without air mycelium, agar plugs were taken from either sector.

## Abbreviations

The following abbreviations are used in this manuscript:

(U)HPLC	(ultra-)high performance liquid chromatography
15ADON	15-acetyl-deoxynivalenol
3ADON	3-acetyl-deoxynivalenol
ACN	acetonitrile
ARS	Agricultural Research Service
b.w.	body weight
bp	base pairs
CONTAM	European Food Safety Authority Panel on Contaminants in the Food Chain
DON	deoxynivalenol
EFSA	European Food Safety Authority
FAO	Food and Agricultural Organization of the United Nations
FMM	Fusarium minimal medium
FUSX	fusarenon-X
IC_50_	half maximal inhibitory concentration
JECFA	Joint FAO/WHO Expert Committee on Food Additives
LC-MS/MS	liquid chromatography-tandem mass spectrometry
LC-MS	liquid chromatography-mass spectrometry
MeOH	methanol
NCBI	National Center for Biotechnology Information
NIV	nivalenol
NMR	nuclear magnetic resonance
NRRL	Agricultural Research Service Culture Collection strain
PCR	polymerase chain reaction
PDA	potato dextrose agar
PHS	pentahydroxyscirpene
PMTDI	provisional maximum tolerable daily intake
QTOF	quadrupole time-of-flight mass spectrometer
TDI	tolerable daily intake
WHO	World Health Organization
